# Challenges in accessing family planning, contraceptive and abortion services, and sexually transmitted infections care during the COVID-19 pandemic in Italy: a mixed method study

**DOI:** 10.1186/s12978-026-02341-w

**Published:** 2026-07-22

**Authors:** Simone Garzon, Alessia Savoldi, Maddalena Cordioli, Ranieri Poli, Igor Toskin, Moazzam Ali, Stefano Uccella, Massimo Mirandola

**Affiliations:** 1 Department of Surgery, Dentistry, Pediatrics, and Gynecology, Unit of Obstetrics and Gynecology, University of Verona, AOUI Verona, Piazza A. Stefani 1, Verona, 37125 Italy; 2 Infectious Diseases Division, Department of Medicine, Verona University Hospital, Verona, Italy; 3 Office 3 - Health Prevention, Research, International Affairs, Communication, Food, Veterinary Medicine and Collegial Bodies, Ministry of Health, Rome, Italy; 4 World Health Organization, Department of Sexual and Reproductive Health and Research, (includes the UNDP/UNFPA/UNICEF/WHO/World Bank Special Programme of Research, Development and Research Training in Human Reproduction [HRP]), Geneva, Switzerland

**Keywords:** COVID-19, Sexual Health Services, Reproductive Health Services, Continuity of Patient Care, Telemedicine, Sexually Transmitted Infections

## Abstract

**Background:**

The Coronavirus Disease 2019 (COVID-19) pandemic disrupted the capacity of healthcare systems. This study aimed to provide a better understanding of the impact of the COVID-19 pandemic on sexual and reproductive healthcare (SRH) services.

**Methods:**

We conducted a mixed-method cross-sectional study at the SRH services of the tertiary care university hospital of Verona, Veneto region, Italy. Questionnaires based on WHO validated tools were administered to all consecutive clients once per month for 11 months (December 2021 to November 2022) to assess SRH services availability and readiness. In-depth interviews (IDIs) have been used to understand participants’ perspectives.

**Results:**

The questionnaire was completed by 100 clients (average age 35.34 years; sexually transmitted infection care service: *n*=49; abortion service: *n*=24, family planning and contraception service: *n*=16, and anti-violence service: *n*=11). All SRH services continued to operate without a shortage of supplies, although some facilities had relocation of staff and a location change. IDIs involved 25 clients at the abortion and family planning services, and 5 clients at the sexually transmitted infection care service. Most clients self-referred to SRH services and sought information online, which was described as helpful, clear, and informative. Remote consultation was the primary access method, allowing planning for in-person attendance after initial triage. This approach was appreciated and considered time-saving and facilitating. Slight delays or longer than anticipated waits were reported by some clients, although mainly in the initial pandemic phase. The relocation of facilities and the restriction of supportive persons were significant barriers to some SRH services. The risk of COVID-19 was perceived as low or less relevant than the reason for accessing SRH care, and it did not limit the decision to access.

**Conclusions:**

Results of the present study reflect the importance of maintaining SRH services for clients during health emergencies. Expanding pre-existing remote services and innovations in SRH care delivery had a key role and were appreciated by clients. Telemedicine for initial triage or online consultations and prescriptions should be implemented in regular practice or predisposed for rapid implementation in emergencies, along with ensuring up-to-date information, particularly online.

## Abstract (plain language summary)

We investigated the impact of the COVID-19 pandemic on sexual and reproductive healthcare (SRH) services, interviewing a group of clients accessing four core facilities: the abortion service, the family planning and contraception service, the center for sexually transmitted infections care, and the center offering support to victims of sexual violence. The study’s results show the importance of maintaining SRH services during health emergencies. The expansion of pre-existing remote services and innovations in SRH facilities implemented during the pandemic played a key role in maintaining services and were appreciated by clients. Online sources of information and the availability of telemedicine were regarded as facilitating access to SRH services and avoiding excessive delays. In contrast, the lack of updates in service delivery changes was one of the main barriers to SRH services utilization.

The investigation of clients’ perception of SRH utilization during the COVID-19 pandemic allowed providing some recommendations:

1. SRH services should be included in the emergency plan as essential services to be maintained.
2. Telemedicine for SRH services through telephone for initial triage or online consultations and prescriptions should be implemented in regular practice or predisposed for rapid implementation in emergencies, because clients appreciated it and avoided unnecessary exposures.
3. In-person care should be continued for selected procedures and vulnerable populations both after triage by telemedicine and by direct access.
4. Ensuring, particularly online, up-to-date information about services accessibility, consultation, and procedures is critical in regular practice and becomes more relevant during emergencies.

## Background

Coronavirus Disease 2019 (COVID-19) was first reported in December 2019 and rapidly spread globally. Three months later, the World Health Organization (WHO) classified COVID-19 as a pandemic threat that spread across all continents [1]. People with clinically significant SARS-CoV-2 infection led to an unprecedented increase in demand for care, with remarkable reallocation of medical resources. These dramatic changes, compounded by the restrictions on movement as part of containment measures, may have led to disruptions in the provision of essential services, including sexual and reproductive health (SRH) care [2–4].

Due to lockdown measures, movement restrictions, fears about COVID-19 exposure, supply chain disruptions of contraceptives, and clinical staff unavailability for SRH services, the United Nations Sexual and Reproductive Health Agency (UNFPA) projected up to 7 million additional unintended pregnancies, particularly in low- and middle-income countries [3, 5]. Models of the potential impact showed that even a 10% reduction in essential SRH services could lead to an estimated 15 million unintended pregnancies, 3.3 million unsafe abortions, and 29.000 additional maternal deaths in 12 months [6–8]. The reduction of routine SRH services, with the cancellation of the “nonessential” visits, and the restriction of laboratory services may have also led to significant challenges in sexually transmitted infection (STI) care, including HIV [9]. The UNFPA also estimated an additional 31 million cases of gender-based violence as a result of lockdown measures lasting six months [5, 10].

Maintaining the continuity of essential health services while keeping people safe during a pandemic is critical for preventing direct and indirect morbidity and mortality [5, 6]. The current study was conducted to address the issue of health service provision continuity and specifically to evaluate the impact of the COVID-19 pandemic on the capacity of the healthcare system to offer SRH services in a third-level hospital in Northern Italy. The main objective was to provide a better understanding of the impact of the COVID-19 pandemic on accessing family planning and contraception service, abortion services, HIV/STI care, and gender-based violence services in developed countries from participants’ perspectives. Although previous studies described the impact of the COVID-19 pandemic on SRH services, most evidence comes from low- and middle-income countries or focuses on single services [11–15]. Our study adds evidence from a high-income European setting and combines WHO-validated facility assessment tools with in-depth interviews across four services. This approach allows providing a comprehensive and user-centered perspective, helping policymakers and health managers to develop policies and strengthen the readiness of SRH services in case of health emergencies.

## Methods

### Study design and setting

The study was carried out in collaboration with the WHO/Human Reproduction Program (HRP) and academic institutions to answer the global research question concerning the impacts of COVID-19 on the capacity of health systems to provide sexual and reproductive health services.

A mixed-method cross-sectional design was used to gather quantitative and qualitative data. The study was conducted at four SRH services of a tertiary care university hospital of the Verona province (Azienda Ospedaliera Universitaria Integrata, AOUI Verona), Veneto region, Italy: the Abortion Service (AS), Family Planning and Contraception Service (FPCS), MISTRA (Multidisciplinare per le Infezioni Sessualmente TRAsmesse), and P.e.t.r.a (Pratiche Esperienze Teorie Relazioni Antiviolenza). The AOUI Verona was selected for the geographical position, which corresponds to one of the areas of the country most severely impacted by the COVID-19 pandemic. Moreover, as the hospital provides all levels of care, it offered extensive opportunities for data collection across various services.

### Population and services

**Fig. 1:**
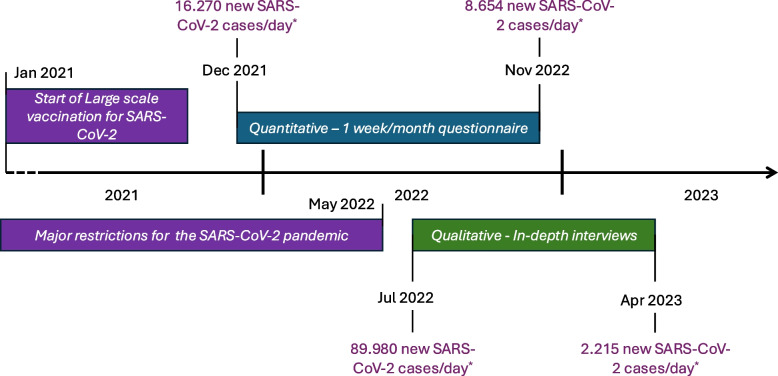
Timeline showing the course of the COVID-19 pandemic during the study period and the timing of quantitative and qualitative interventions

The population of the Verona province is 922.383 individuals, and at the study’s beginning in December 2021, 119.250 SARS-CoV-2 cases had been confirmed [16, 17]. A timeline showing the course of the COVID-19 pandemic during the study period, along with the timing of quantitative and qualitative interventions, is provided in Figure 1.

The AS at the Unit of Obstetrics and Gynecology of the AOUI Verona is the leading center providing abortion care service in the Verona Province. Women seeking for abortion are referred to our center from the primary care centers of the Verona Province. The AS is a physician-led public service that offers counseling for unplanned pregnancy and abortion treatment and provides medical and surgical abortion, all paid for by the National health care system.

The FPCS for the general population is provided at multiple centers in the Verona province. At the Unit of Obstetrics and Gynecology of the AOUI Verona, counseling for people seeking contraceptive information and prescription or couples planning a conception is provided by the obstetrics and general gynecologic services together with a gynecologic assessment.

The MISTRA center targets both general and key/vulnerable populations (MSM, transgender people, sex workers, adolescents and young adults, etc.) [18]. This public service offers tailored counseling and testing for HIV and other STIs, as well as the combined preventive approach and linkage-to-care and follow-up for chronic infections. The service is a primary care service with direct access for clients.

The P.e.t.r.a. center is a public service provided by the Municipality of Verona, which offers services, advice, and support to men and women of any age who have been raped or sexually assaulted or have been a victim of domestic violence.

### Qualitative methods: In-depth interviews

The study used in-depth interviews (IDIs) to collect qualitative data and understand participants’ perspectives of SRH service availability, readiness, and barriers to services.

IDIs were semi-structured and administered, in-person or virtually, following specific topic guides by study investigators who were not directly involved in the clinical activity, per the pandemic trend and clients’ preferences. Topic guides were used to gather information on 1) Knowledge about COVID-19 (such as cause, signs, and symptoms); 2) Care seeking experience during COVID-19 waves and perception of risk of SARS-CoV-2 infection; 3) Concerns about potential reproductive health issues caused by COVID-19; 4) Needs relating to SRH services according to the service accessed by the client; 5) Barriers and health-seeking behavior for accessing SRH services. The topic guides were validated by an international and multidisciplinary panel of experts (e.g., the WHO Regional and Country Offices, UNFPA Country Office, Research Centre for Public Health, Universities, and National Research Institutes). The core research team and local facility collaborators reviewed the topic guides and made local adaptations. The original questions were translated into Italian and then back-translated into English to ensure no discrepancies. Thereafter, a piloting phase of the IDIs topic guide was carried out before the official start of the study. IDIs were conducted by members of the local study team, all trained in collecting qualitative data. For virtual IDIs, additional training was conducted only for those using the virtual platform. Privacy, confidentiality, and anonymity were guaranteed for all study participants who could deactivate the video in the case of virtual IDI.

All consecutive clients who accessed (virtually or in-person) targeted SRH services were assessed for eligibility. The inclusion criteria were being 18 years or older and having the ability to read and speak Italian fluently. Exclusion criteria were refusal to participate or provide written informed consent. All clients with significant distress or safeguarding concerns were not considered for the study.

Consenting clients were asked to complete a brief demographic questionnaire before interviews to monitor sample variation. A purposive sampling of up to 10 clients per SRH service was planned at baseline (December 2020-April 2021) and endline (April 2022-November 2022). Different clients were planned for baseline and endline due to the ethical sensitivities of the topic areas for discussion. For the MISTRA center and FPCS, a potential sample of 5 partners per SRH service was planned. However, due to organizational issues, IDIs were collected only between July 2022 and April 2023. Another major problem was the availability of clients to be interviewed after the required period that had to pass between service utilization and interview administration. Most users declined their availability after the initial consent. This was mainly due to a specific desire to avoid living again the consultation/procedure experience. The enrolment procedure was slightly changed to allow willing clients to be interviewed while at the facility to minimize the participation issue.

### Quantitative methods: assessment of health facility availability and readiness tools

A questionnaire was developed to gather information on the availability and readiness of SRH services during the emergency response to COVID-19. The instrument was conceived based on five already existing validated tools: 1) WHO Service Availability and Readiness Assessment Guide [19]; 2) WHO Health Emergency Facility Assessment Checklist [20]; 3) WHO safe abortion assessment tool [21]; 4) WHO Gender-based Violence Health System Readiness tool [22]; 5) WHO STI assessment tool [23]. The local research team adapted the questionnaire according to Country legislation and the type of service provided (*i.e*., existing policies, SRH treatment guidelines, staffing norms and standards, types of facilities, national essential medicines lists, and national health information systems). Finally, the questionnaire form was piloted internally and fine-tuned, translated into Italian, and then back-translated into English. No discrepancies were found between the two versions, and the adaptation was satisfactory. A paper version of the questionnaire was used by clinical staff and then regularly handed over in bunches on a predefined day of the week to a research assistant for the data entry into the WHO clinical trial management system (WHO OpenClinica).

The assessment of health facility availability and readiness tools was administered as a survey to all consecutive eligible clients who accessed, virtually or in-person, each SRH service during one predefined week of each month. This consecutive sampling strategy was applied over the entire 11-month study period (December 2021 to November 2022). The study enrollment of all attending clients was based on standard operating procedures (SOP) cleared by the Ethics Board. The participation was voluntary, and services were provided equally to consenting and non-consenting clients; therefore, not all eligible clients completed the questionnaire. Inclusion criteria were 18 years or older and the ability to read and speak Italian fluently. Exclusion criteria were the refusal to participate and provide informed consent. Data entry was carried out by a designated data entry operator/clerk who then entered the information from the questionnaire into Open Clinica. Data was then double-entered as a quality assurance procedure by the same data entry clerk, only after the official closure of data collection.

### Data analysis

The qualitative analysis represented the principal analysis of the study. Audio-recorded data were transcribed verbatim and de-identified using identifier (ID) numbers (name/location). All interviews were conducted in Italian. All interviews were analyzed by the core research team supported by the University of Brighton. Analyses followed the general approach of content analysis, including reading through the verbatim transcriptions and notes to understand what was being expressed by the participants, noting down emerging themes and patterns in the data, and condensing the data. This analytical approach allowed themes to be identified both inductively (unanticipated findings arising from the interviews) and deductively (based on the project objectives). The researchers decided on the unit of analysis and creation of units of meaning, which have been condensed into codes while ensuring the core meaning is not lost or distorted. The codes were then used to generate categories (and subcategories, when required) and condensation of related categories into themes that convey the meaning of the data. The researchers and the project coordinator systematically discussed the progress of the coding method. The NVivo qualitative analysis software (version 1.7.1) supported the analysis. Two researchers analyzed the same data set and were guided by general questions, specific questions, and comparisons. The data were analyzed according to the steps suggested by Elo & Kyngäs [24].

Descriptive statistics were applied to summarize quantitative data, which were meant to provide basic information about the characteristics of clients accessing the health facilities, as appropriate. Given the achieved sample size, an in-depth analysis of the data at the SRH service level was impossible to perform, and any meaningful conclusion of statistical significance could not then be drawn. Anonymized data were securely submitted to WHO via Open Clinica. WHO was responsible for cleaning the local anonymized dataset and performing the data analysis. For quantitative data analysis, STATA Version 17.0 was used (College Station, TX: StataCorp LP), while SAS version 9.4 (SAS Institute Inc., Cary, NC, USA) was used for data management.

## Results

### Quantitative component of the study

All four healthcare facilities continued to provide services during the pandemic.

During lockdown periods, the AS did not experience any major issues or barriers to ensuring continuity of care. No cases of referrals to other healthcare facilities were reported, and no shortage of drugs, supplies, and equipment for safe abortion care was experienced. National abortion guidelines and safe abortion checklists/job aids were available in the facility, and staff had received continuous training in safe abortion and related procedures as part of the “continuous education in medicine” as per national requirements for all health professionals. Instead, the COVID-19 pandemic partially impacted the FPCS and MISTRA center opening times due to the relocation of medical doctors and nurses, consequently increasing appointment waiting times. Moreover, the MISTRA center changed location several times in a few months because of the COVID-19 treatment facility reorganization. However, at both SRH services, national guidelines and safe checklists/job aids remained available in the facilities, and staff had received continuous training as part of the “continuous education in medicine” as per national requirements for all health professionals. No reproductive health medicines or commodities (i.e., different contraception methods) were out of stock. Similarly, the MISTRA centre continued to offer HIV rapid testing. Serology for syphilis, hepatitis A, B, and C, and HIV, Nucleic Acid Amplification Test (NAAT) for Neisseria gonorrhoeae, Chlamydia trachomatis, and Mycoplasma genitalium, together with other microbiological assessments (i.e., bacterial and fungal culture examination), continued to be performed at the facility and sent to the hospital internal laboratory for analysis. There were no shortages of drugs, both in the hospital and community pharmacies, for the treatment of STIs during the time periods considered by the study. The P.e.t.r.a. center remained open for 24 hours with no restrictions during the pandemic; therefore, the center continued to provide the same level of care during the pandemic.

At baseline (December 2020-April 2021), an average of 45 monthly clients visited the AS, 35 the FPCS, 135 the MISTRA, and 53 the P.e.t.r.a. center. At the endline (and April 2022-November 2022), an average of 47 monthly clients visited the AS, 36 the FPCS, 190 the MISTRA, and 11 clients the P.e.t.r.a. center (Table 1).

**Table 1:** Distribution of participants across SRH services

SRH Service	Baseline monthly attendance (average)	Endline monthly attendance (average)	Questionnaire respondents (n)	IDIs (n)
Abortion Service (AS)	45	47	24	10
Family Planning and Contraception Service (FPCS)	35	36	16	15
MISTRA	135	190	49	5
P.e.t.r.a.	53	11	11	0

*SRH* Sexual and reproductive healthcare, *MISTRA* Multidisciplinare per le Infezioni Sessualmente TRAsmesse, *P.e.t.r.a* Pratiche Esperienze Teorie Relazioni Antiviolenza, *IDIs* in-depth interviews

Overall, 100 clients across the four facilities completed the general questionnaire during the set week of each month (Centro MISTRA: *n*=49; AS: *n*=24, FCPS *n*=16, and P.e.t.r.a.: *n*=11; Table 1). The average age of clients was 35.34 years (Standard deviation [SD] 9.31; min 19 – max 62) with no statistically significant difference across study sites. Table 2 summarizes socio-demographic characteristics. In all cases, the service type used mirrored the service accessed. Having difficulties or problems with partners was reported by all P.e.t.r.a. clients and by 28.6% and 4% of FPCS and MISTRA respondents, respectively. No clients reported issues with partners at the abortion service (p≤.001). Respondents’ marital status varied largely across services: 73.47% and 54.55% of MISTRA and P.e.t.r.a. clients, respectively, described themselves as single, whereas amongst the abortion and FPCS, the majority were married/cohabiting (57.89% and 76.17% respectively). These differences may reflect distinct motivations for accessing SRH services (p≤.001); in fact, the main reason for accessing the MISTRA center was related to HIV/STI counseling and screening. These data refer to the entire study period and were not stratified by baseline and endline due to the limited sample size per service.

**Table 2:** Characteristics of participants who completed the general questionnaire (*N*=100)

**Characteristic**	
Gender	
Female	53 (53)
Male	47 (47)
Mean number of years in education (range)	14.5 (5–24)
Relationship status	
Single	50 (50)
Cohabiting	40 (40)
Separated	7 (7)
Divorced	3 (3)
Ethnicity	
White European	74 (74.7)
Han	3 (3)
African	4 (4)
South Asian	2 (2)
East Asian	1 (1)
Latino/Hispanic	9 (9.1)
Middle Eastern	6 (6.1)
Currently pregnant	
Yes	20 (37.7)
No	33 (62.3)
No. of pregnancies	
0	17 (32.1)
1	10 (18.9)
2	17 (32.1)
3	4 (7.5)
4	3 (5.7)
5	2 (3.8)
No. of abortions	
0	34 (64.1)
1	15 (28.3)
2	3 (5.7)
3	1 (1.9)
No. of births	
0	27 (50.9)
1	14 (26.4)
2	9 (17)
3	0 (0)
4	3 (5.7)
No. of living children	
0	26 (49.1)
1	15 (28.3)
2	10 (18.9)
3	0 (0)
4	2 (3.8)

### Qualitative component of the study

IDIs were performed for 25 clients at the AS and FPCS and for five clients at the MISTRA center (Table 1). No clients were interviewed from the P.e.t.r.a. center, as eligible clients declined participation or were unavailable. No partners were involved due to the unwillingness of clients to involve their partners. All IDIs were conducted in person. The socio-demographic characteristics of the 25 interviewees at the AS and FPCS are summarized in Table 3.

**Table 3:** Characteristics of participants who underwent In-Depth Interviews

**Characteristic**	**Abortion service**	**Sexual Health and Contraception Service**
Mean age and (SD) range	30.3 (7.45) 20–39	30.6 (4.38) 27–40
Gender		
Male	0	4
Female	10	11
Sexual orientation		
Heterosexual	9	8
Gay man	0	3
Bisexual	0	2
Pansexual	0	0
prefer not to say	1	2
Relationship status		
Single	2	3
Living together (unmarried)	3	3
Partner (not living together)	4	8
Married	1	1
Polyamorous relationship	0	0
Ethnicity		
European	10	15
Religion		
None (or agnostic)	2	8
Christian	8	7
Muslim	0	0
Other - spiritualist	0	0
Education		
GCSEs/O-Levels/school until 16	1	0
A-Levels/equivalent/school until 18	2	2
Undergraduate degree	0	5
Postgraduate degree	0	8
Professional qualification	5	0
None	2	0
Employment		
Part-time employment	2	0
Full-time employment	4	3
Self-employed	1	2
Maternity leave	0	1
Unemployed	2	1
Retired	0	0
Full-time student	1	8
Number of times pregnant		
0 (or N/A)	0	9
1	5	1
2	2	0
3	3	0
4+	0	0
Number of children		
0	6	14
1	4	1
2	0	0
3	0	0

#### Knowledge of COVID-19, risk, and prevention

Participants showed a good understanding of COVID-19-related knowledge, including symptoms, transmission pathways, and prevention strategies. Mask-wearing and hand hygiene were generally considered the easiest and most efficacious measures to be adopted for limiting the SARS-CoV-2 spread. The level of engagement with preventative behaviors broadly varied according to the different waves of the pandemic and the related governmental dispositions. Nevertheless, most participants revealed that they continued to wear surgical masks in crowded areas despite being considered optional: “*Wearing PPE as, for example, the mask, frequently washing hands and airing the room (MISTRA, July 2022).”*

Most participants reported accessing governmental and other institutional websites to find information on COVID-19; these “official” sources were viewed as providing the most accurate, reliable, and up-to-date information. Further sources of COVID-19 information included news channels and websites (*e.g.,* SkyNews, National Newscast) and the most relevant national newspapers (*e.g.,* La Repubblica, Il Corriere della Sera). All participants stated that they did not trust social media sites, such as Facebook, Instagram, and Twitter, to retrieve information on COVID-19 because they were considered unreliable information sources, which could dangerously disseminate wrong messages, raising unfounded worries among individuals. Workplaces, family and friends, and healthcare providers (e.g., General Practitioners) were additional sources of information on COVID-19. Many participants perceived themselves at moderate-low risk of COVID-19 infection: *“...At work, we all wear masks –always, every day- so I think it’s low (FPCS, February 2023).”*

#### Psychosocial impact of the COVID-19 pandemic

Many participants described the negative impact that COVID-19 and government restrictions had on their mental health and well-being. Almost all participants were negatively affected by home isolation and the forced working at home requirement. Some participants reported suffering from financial concerns associated with job uncertainties. No participants reported a need for professional support for their mental health. However, most participants noticed and benefited from increased support from and to family and friends.

Interestingly, some participants disclosed that the COVID-19 pandemic also had a positive impact at the personal level. The pandemic led to relevant positive changes, including stronger relationships with family members, an opportunity to reflect on themselves, and a focus on reassessing life priorities. “*I had a chance to stay longer with my parents and my sister; we were all secluded, and no one worked (out-of-home) (MISTRA, January 2023).”*

#### Initial information seeking and access to the service

Most clients self-referred to the AS (clients reported that the decision to terminate the pregnancy was made either by themselves or jointly with their husbands/partners); a few were referred by their gynecologist: *“Actually, I was advised by my gynecologist, and then I came to you” (AS, February 2023).* Conversely, all participants reported accessing the MISTRA center and FPCS directly, based on their own decisions, without going through their general practitioner or other healthcare service/provider. For most participants, this was because dedicated SRH clinics were their usual point of access for their sexual health and contraceptive needs. Indeed, most MISTRA center participants reported having accessed the service more than once during COVID-19 (*i.e.,* since March 2020), with some having accessed the service regularly and as frequently as every three/six month for the follow-up of chronic infections or monitoring of therapies for infectious diseases.

Most participants tried to find online information about the hospitals in the area offering pregnancy termination services. The process was described as easy and straightforward, as the Verona Hospital is the leading provider in the area: “I *searched the internet and then I spoke with my doctor […] who gave me guidelines on how to move (AS, March 2023).”* Similarly, participants (regular and less regular clinic attendees) sought online information about the MISTRA center and FPCS before accessing them. They typically described this web-based information as helpful and self-explanatory. Finding information about the MISTRA center and FPCS provided by the clinic was described as easy and quick, with just one participant reporting that *“I had some trouble finding the phone line free (MISTRA, March 2023).”*

#### Patient journey’ – the process of seeking and receiving care

Concerning the AS, participants called the clinic to confirm the initial information received from general practitioners, gynecologists, or found at other sources, and arrange the first appointment with the doctor. One participant reported a slight delay in the care pathway between the initial consultation and the procedure. Participants also noted that information was shared during telephone consultations with the health care provider about the types of treatment available, and this was seen favorably by the users as the consultation time was reduced. The service also offers termination of pregnancy aftercare (*e.g.,* medical check-ups, psychosocial support) if required or wanted, and during COVID-19, aftercare was mainly provided *via* telephone.

Participants reported accessing the MISTRA center and the FPCS in various ways, including telephone consultations, but mainly in-person clinic attendance, and, more rarely, getting online prescriptions. Participants reported being triaged for in-person appointments when booking or after telephone consultations.

#### Perception of risk of (or safety from) COVID-19 infection during care-seeking

Interviewees did not consider the risk of COVID-19 infection when deciding whether to access the AS. The perception that COVID-19 infection risk was lower at the abortion clinic compared to other healthcare settings played an essential role in the participant’s mind. In addition, the urgency to access the procedure on time counterbalanced the fear of contracting COVID-19 and the perception of SARS-CoV-2 infection risk. *“No one changed my appointment; I was the one who preferred to move it (AS, July 2022)”.* Similarly, most of the participants did not consider the risk of COVID-19 infection when accessing sexual health or contraception services at the clinic, as they felt that proper precautions were in place to minimize risk. One participant who accessed the FPCS during the 2nd lockdown described how COVID-19 was no longer a consideration or concern for her. *“I wasn’t worried about COVID; the appointment was scheduled, and there would be just a few people in the room (FPCS, March 2023).”*

All participants described a range of safety measures that the services had implemented to reduce the spread of COVID-19, including mask-wearing by clients and staff, hand sanitizer availability, and body temperature check at reception, spacing between chairs in the waiting area, as well as receiving a text message before their appointment informing them to stay at home if they had any symptoms of COVID-19. *“All necessary measures have been used [...] disposable gloves, FFp2 mask. (FPCS, March 2023)”.* None of the participants complained about the restriction of companions/supportive persons at the clinic.

#### Barriers and facilitators to receiving care

Initial contacts and consultations with health care providers were conducted remotely over the telephone in all SRH services. Most participants considered that remote appointments facilitated access to the service by reducing the time spent traveling, which was especially important to those who worked or had to organize childcare during the early stages of the pandemic when in-person appointments were limited. Several participants found speaking to someone over the phone more comfortable than in person.

Delays in access to the AS (or longer than anticipated waits) were feared by patients reemerging the delays during the first epidemic wave; however, none have complained of significant distress due to delays. *“In fact, I feared there would be long waits instead; it was all pretty quick (AS, March 2023).”* Visitors were restricted throughout the pandemic (up to April 2022); therefore, the accompanying person was allowed only for a few clients with serious clinical reasons. This restriction was reported to have a negative impact on the care experience of clients who attended the AS, although none complained about this restriction.

Regarding the MISTRA center and FPCS, there were mixed experiences regarding appointment availability depending on when participants were trying to access the service in relation to the pandemic trend and associated restrictions. The length of the waiting list strongly depended on the epidemic wave. The most extended delay was registered during the first pandemic wave due to personnel redeployment and patient accrual. The first lockdown (March to June 2020) was described as challenging by several participants, particularly for MISTRA clients, as being the most difficult to access, as the clinic changed location several times in a few months because of COVID-19 treatment facility reorganization. *“During lockdown*(*1*^*st*^* one), the clinic has been moved three times, unannounced [...]. I went all over the hospital and then asked who was available (referring to medical staff). (MISTRA, November 2022).”* In this regard, some participants noted that triaging by telephone ensured that in-person appointments were given to those who needed to be seen, which reduced waiting times (both for an appointment and when at the clinic).

## Discussion

### Principal findings

Quantitative findings showed resilient SRH services during the study period. All four services continued to provide care, without major issues or barriers, and only minor changes, such as location. Qualitative results confirmed these observations and provided further insights into the perception of clients, highlighting strengths and limitations of SRH services as well as details on interactions with patients. Clients sought online information about the SRH services, which were typically described as helpful, clear, and informative. Most participants, across both facilities, self-referred to services as per the usual access route. Remote consultation was the primary strategy for accessing SRH services, enabling planning for in-person attendance after initial triage. Most clients appreciated this approach, considered it time-saving, and found it facilitated access. Slight delays or longer than anticipated waits were reported by some clients, but not for all services, particularly in the initial phase of the pandemic. The relocation of the clinic facilities during lockdown periods and the restriction of supportive persons were significant barriers to some SRH services. The risk of COVID-19 was perceived as low because of the comprehensive infection control measures implemented, which did not limit the decision to access SRH services. Although these findings suggest resilient SRH services with appropriate strategies, the characteristics of patients who underwent IDIs and the exclusion of non-Italian speakers question whether these observations apply to vulnerable populations or minorities. The absence of clients from P.e.t.r.a. for qualitative data confirms the difficulties in investigating the vulnerable population, which deserve dedicated strategies.

### Interpretation

Study results align with previous research on how COVID-19 has disrupted and impacted SRH services [11, 12]. All facilities included in our study continued to operate throughout the pandemic, and most clients could access the SRH care they needed with limited delays, indicating resilience in service delivery. The difficulties experienced by the MISTRA center due to staff redeployment in COVID-19 wards as part of the Infectious Disease Unit are consistent with a drastic decrease in all routine services, including HIV follow-up visits, HIV and STI testing, and HIV-pre-exposure and post-exposure prophylaxis management reported by a United States survey [25].

The rapid implementation of telemedicine was one of the most significant adaptations experienced by SRH services. Clients appreciated the availability of information about SRH services online and the remote consultation or initial contact and assessment by telephone, which was identified as facilitating SRH access. The key benefits of telehealth identified by participants align broadly with those described in the literature, including safety (*e.g.,* minimizes the risk of COVID-19 infection), privacy, convenience, and access. Conversely, limitations of telehealth, such as the challenges associated with the inability to have a physical examination or the difficulty in communicating without body language, were not reported by clients [26, 27].

Clients showed a good understanding of COVID-19-related knowledge, including symptoms, transmission pathways, and prevention strategies. Remarkably, the application of safety measures by SRH services was appreciated by clients and was the main reason for considering the risk of COVID-19 marginal and not affecting the choice to access the services. In this regard, one exception was represented by the pregnancy termination, which was frequently regarded as urgent and more relevant than the risk of COVID-19, which was not considered at all. Clients accessing the AS appreciated safety measures, but COVID-19 risk was, in any case, considered less relevant than pregnancy termination. This aspect may be unique to Italy, given abortion can be requested based on a personal choice of the woman only up to 90 days of pregnancy (Law n. 194 - May 22, 1978). Time constraints represent a potential source of stress for clients needing access to SRH services; the fear of delays was frequently reported by clients accessing the AS, although rarely experienced.

Interviewed clients who experienced a few barriers to accessing SRH services. The main obstacle was obtaining updated information on the SRH service activity. Changes in the availability of the service and its location without appropriate communication to clients were some of the more stressful factors reported.

Our findings underlined the importance of implementing digital technologies in healthcare practice to support physical distancing during the COVID-19 pandemic. The growing utilization of telemedicine was positively valued by clients during the pandemic, especially those accessing the MISTRA center. The literature widely described the benefits of telemedicine in several SRH fields. Concerning abortion, a study by Awowole et al. showed that telemedicine not only facilitates access for women seeking abortion services but also provides the additional benefits of confidentiality and avoidance of stigmatization, with similar outcomes to facility-based management [28]. Telemedicine also helps bridge gaps in contraceptive care and HIV-STI testing; a systematic review of randomized controlled trials including 5400 individuals has shown that sexual telehealth interventions among adolescents were found to increase self-efficacy of condom use and being tested for STIs [29]. Beyond the pandemic, the added value of telemedicine in clinical routine practice has been recognized by several countries, which have facilitated the implementation and the scale-up of telehealth services by introducing regulatory flexibilities and incentives to foster implementation with coordination from providers and technology companies [30].

All these observations align mainly with previous evidence from other high-income countries. Completely different is evidence from low and middle-income contexts, where the COVID-19 pandemic created substantial challenges in accessing SRH services [13–15]. Although in some settings the implementation of digital health interventions mitigated service disruptions [31], SRH services availability was often impaired by movement restrictions, facility closures, stock-outs of contraceptives, and reduced health workforce availability [13–15]. In some low and middle-income countries, the COVID-19 pandemic exacerbated pre-existing inequities in SRH service access [13–15, 32]. Therefore, our findings must be interpreted in function of their geographical limitation, as a representation of high-income contexts. Similarly, immigrants and adolescents are underrepresented, impeding to know whether for these subgroups SRH services remained adequate. Notably, both characteristics, alone or combined, have been reported to be associated with a lower-than-expected SRH care utilization during the COVID-19 pandemic [33–35].

### Strengths and limitations

This mixed-methods study provided an in-depth insight into clients’ perception of health facility availability and readiness to deliver SRH services during the COVID-19 pandemic. Semi-structured IDIs conducted by expert and trained research staff strengthen the value of the obtained information. Moreover, the robust methodology applied to record, translation, and qualitative analysis of the IDIs strengthens their value. However, in line with qualitative methods, caution should be exercised in considering the transferability of findings beyond the context in which the data were collected. In this regard, quantitative data on the characteristics of the study population allow us to clarify which populations our results may apply to. Furthermore, among limitations, we recognize that some groups of participants were not recruited as per the protocol, such as non-Italian speakers, and the lack of participants enrolled at the first-time point for the IDIs may have missed the most affected during the pandemic. We were also unable to recruit clients from the P.e.t.r.a., so there is a lack of qualitative data generated by clients of that facility. In this regard, the main reason was the refusal to participate, highlighting the natural difficulties in investigating such vulnerable populations. Finally, the sample’s limitations are present, like the limited number of clients of the MISTRA center. On these bases, we recognize that the representativeness of qualitative findings is limited. Results can be considered reliable for services, such as the AS and FPCS, whereas comments on the MISTRA service should be made with more caution.

A further point of limitation by study design is that all client participants had accessed care or treatment; therefore, our sample may include participants with biased experiences of care (more positive) and exclude participants who were unable to access the services or experienced more barriers. Moreover, the demographic characteristics of interviewed clients, along with the exclusion of non-Italian speakers, suggest that some of the vulnerable people (e.g., immigrants, young people, and those with chaotic lifestyles) are underrepresented. This finding has been supported by other United Kingdom studies looking at SRH access [36, 37]. One reason is that vulnerable people may have found it particularly difficult to access SRH during the pandemic. However, for some SRH services, such as the AS, our observations suggest that the presence of clients unable to access due to the pandemic is unlikely, strengthening the representativeness of IDIs.

## Conclusions

The results of this study reflect the importance of maintaining SRH services during health emergencies. The expansion of pre-existing remote services and innovations implemented during the pandemic had a key role and was appreciated by clients. Online sources of information and the availability of telemedicine were regarded as facilitating access to SRH services and avoiding excessive delays. In contrast, the lack of updates in service delivery changes was one of the main barriers to SRH services utilization. The investigation of clients’ perception of SRH utilization during the COVID-19 pandemic allows providing some recommendations: 1) SRH services should be included in the emergency plan as essential services; 2) telemedicine for SRH services through phone for initial triage or online consultations and prescriptions should be implemented in regular practice or predisposed for rapid implementation in case of emergency because appreciated by clients and avoiding unnecessary exposures; 3) in-person care should be continued for selected procedures and vulnerable populations both after triage by telemedicine and by direct access; 4) ensuring, particularly online, up-to-date information about services accessibility, consultation options, and procedures is critical in regular practice and becomes more relevant during emergencies. However, we recognize that these recommendations apply to high-income contexts and are limited to the represented population. Further research focusing on vulnerable populations, such as adolescents and language minorities, is warranted better to address SRH services readiness for the entire population and define which interventions really increase and guarantee appropriate SRH care.

## Data Availability

The data will be available upon request as per the WHO policies. Requests for access to data can be sent to alimoa@who.int.

## Abbreviations

COVID-19: Coronavirus Disease 2019
WHO: World Health Organization
SRH: Sexual and reproductive health
UNFPA: United Nations Sexual and Reproductive Health Agency
STI: Sexually transmitted infection
AOUI Verona: Azienda Ospedaliera Universitaria Integrata
AS: Abortion Service
FPCS: Family Planning and Contraception Service
MISTRA: Multidisciplinare per le Infezioni Sessualmente TRAsmesse
P.e.t.r.a: Pratiche Esperienze Teorie Relazioni Antiviolenza
